# Comparative analysis of stress markers, metabolic health, and gut microbiota in healthy and disabled dogs in long-term shelters in Thailand

**DOI:** 10.1371/journal.pone.0344383

**Published:** 2026-03-13

**Authors:** Adul Saengthong, Janine L. Brown, Patcharapa Towiboon, Khanittha Punturee, Songphon Buddhasiri, Korakot Nganvongpanit, Veerasak Punyapornwithaya, Wuithipong Tocharoennirattisai, Jaruwan Khonmee

**Affiliations:** 1 Faculty of Veterinary Medicine, Chiang Mai University, Chiang Mai, Thailand; 2 Smithsonian National Zoo & Conservation Biology Institute, Front Royal, Virginia, United States of America; 3 Center of Elephant and Wildlife Health, Chiang Mai University Animal Hospital, Chiang Mai, Thailand; 4 Cancer Research Unit of Associated Medical Sciences (AMS-CRU), Faculty of Associated Medical Sciences, Chiang Mai University, Chiang Mai, Thailand; Wageningen Universiteit, NETHERLANDS, KINGDOM OF THE

## Abstract

Regular welfare assessments are essential for identifying and addressing the physical, behavioral, and emotional needs of shelter dogs, thereby ensuring their well-being and improving chances for adoption or long-term stability within the shelter environment. This study compared biomarkers of physiological stress [fecal glucocorticoid metabolites (fGCM)], metabolic status [serum malondialdehyde (MDA), glucose, triglycerides, low- (LDL) and high- (HDL) density lipoproteins], and fecal microbiota composition over a 1-month period in healthy and spinal-injured disabled dogs housed in a large dog rescue shelter in Thailand for over 1 year. No significant differences in fGCM concentrations were observed between groups, indicating comparable levels of physiological stress in healthy and disabled dogs under long-term shelter conditions. In contrast, disabled dogs exhibited significant metabolic and oxidative alterations, including elevated MDA and glucose, along with lower triglyceride and HDL concentrations. Microbial analyses revealed comparable alpha diversity but differences in beta diversity between groups. Notably, disabled dogs exhibited a reduced Firmicutes-to-Bacteroidetes (F/B) ratio and decreased relative abundances of beneficial taxa such as *Peptostreptococcaceae*. These findings suggest that, despite comparable hormonal stress indicators, disabled dogs may experience subclinical physiological shifts that warrant more nuanced welfare monitoring. A multifactorial assessment incorporating metabolic and microbial parameters is recommended to ensure comprehensive welfare evaluation for physically impaired shelter dogs.

## 1. Introduction

The global population of stray and abandoned dogs has increased significantly in recent years, driven by rapid urbanization, uncontrolled breeding, and shifts in pet ownership patterns [1,2]. As a result, animal shelters have become increasingly important in providing temporary or long-term refuge for lost, abandoned, or surrendered dogs, offering basic care, medical treatment, and opportunities for adoption [3]. Beyond rehabilitation and rehoming, many shelters also care for dogs that are unlikely to be adopted, such as older dogs, dogs with chronic illness, or dogs with physical disabilities, providing them with a safe and structured environment [4]. Despite this critical function, shelters often face challenges in maintaining optimal welfare standards, as limited resources and high population density can affect both physical and psychological well-being of animals in their care [5]. While the exact number of animal shelters in Thailand is unknown, anecdotal sources suggest there may be over 90 government and private shelters in the country, and another 40 temples that care for stray dogs, now estimated at over 1.6 million animals [6].

While shelters aim to provide safety and care, the environment itself may be inherently stressful for dogs. Prolonged exposure to confinement, high noise levels, limited social interactions, and frequent changes in routines or caregivers can lead to psychological distress [7]. Studies have shown that shelter dogs often exhibit behavioral signs of distress, such as excessive barking, stereotypic pacing, withdrawal, or aggression [8,9]. These behaviors are frequently accompanied by physiological changes, including elevated cortisol concentrations, altered heart rate variability, and suppressed immune function [10]. Chronic stress not only compromises animal welfare but can also hinder recovery from illness or injury and reduce the likelihood of successful adoption [7].

Given the significant impact of stress on shelter dogs, it is important to employ reliable, comprehensive welfare assessment methods that integrate both behavioral and physiological indicators [11]. Among the physiological measures, glucocorticoid (GC) concentrations, particularly cortisol and its metabolites, are especially valuable for evaluating factors related to animal welfare [12,13]. GCs are released via activation of the hypothalamic-pituitary-adrenal (HPA) axis in response to stressors and are closely linked to both acute and chronic stress states [14,15]. Measurement of GCs can be conducted invasively using blood samples or noninvasively in feces, urine, and saliva [15]. Because blood and salivary cortisol fluctuate rapidly in response to acute stress, these measures may have limited value for assessing long-term physiological stress in a shelter setting. In contrast, monitoring fecal GC metabolites (fGCM) provides a more suitable non-invasive indicator of chronic stress, reflecting integrated physiological responses without causing additional disturbance to the animals [12,13,16].

In addition to fGCM, analysis of gut microbiota composition can also serve as a useful indicator of welfare status [17]. A growing body of research has demonstrated that psychological and environmental stressors can induce significant changes in the diversity and structure of the gut microbiome, which, in turn, influence physiological and behavioral health outcomes [16,18]. For example, in dogs, one study found that the gut microbiota of anxious animals had increased *levels of Lactobacillus* spp., *Bifidobacteria*, and *Enterobacteriaceae* compared with healthy counterparts [19], whereas others reported altered microbiome and metabolome profiles in fearful [20] and aggressive [21] dogs. There is increased interest in understanding relationships within the gut-brain axis across other species, both wild and captive. For example, in red squirrels, ecological factors like food availability and population density independently influenced both GCs and microbial diversity. Higher GCs were associated with lower gut microbiome diversity and an increase in taxa associated with metabolic function, whereas gastrointestinal pathogens decreased with increasing GCs [22]. Thus, integrating GC metabolite analysis with gut microbiota profiling can provide a robust, non-invasive framework for evaluating stress and welfare in shelter dogs.

Other physiological indicators include insulin, glucose, and fructosamine, which are fundamental for assessing glycemic control and identifying metabolic stress or dysfunction [23–25]. Blood lipid profiles, such as triglycerides, cholesterol, low-density lipoprotein (LDL), and high-density lipoprotein (HDL), further provide snapshots into metabolic health, energy regulation, and cardiovascular risk [26,27], while MDA serves as a key indicator of oxidative stress and lipid peroxidation, reflecting cellular damage caused by reactive oxygen species [28–30]. Together, these measures can offer a comprehensive picture of metabolic and physiological health, supporting early detection of stress-related dysfunction and the monitoring of interventions aimed at improving canine welfare and overall well-being.

Among shelter-housed animals, dogs with physical disabilities such as amputations, paralysis, visual or auditory impairments represent a particularly vulnerable group. These animals often require specialized care and may not be properly socialized with other dogs, and thus face barriers to adoption [31]. Despite their increasing presence within shelters, the welfare of disabled dogs remains understudied, particularly in terms of how well they cope with environmental or husbandry stressors compared to healthy counterparts. A better understanding of stress responses in shelter dogs with disabilities versus those without is therefore essential for improving shelter management, enrichment practices, and developing individualized care protocols. Thus, this study compared health and welfare biomarkers between healthy and disabled dogs at a shelter in Thailand as part of a broader investigation into how management practices and welfare conditions affect behavior and health indicators [32]. We hypothesized that disabled and healthy dogs would not differ in physiological stress markers, metabolic profiles, or gut microbiota composition under long-term shelter conditions with the same management. By evaluating fGCM, we sought to determine whether disabled dogs experience stress levels comparable to those of healthy dogs under the same standardized shelter conditions. A comparison of metabolic markers and gut microbiota characteristics could further help determine whether disabled dogs exhibit metabolic and microbial alterations associated with oxidative stress and limited mobility under chronic physical impairment. The findings of this study are expected to inform the development of tailored management approaches to support the welfare of both disabled and healthy dogs living in animal shelters.

## 2. Methods

### Ethical consent

This study was approved by the Faculty of Veterinary Medicine, Chiang Mai University (CMU) Research Ethics Committee (R22/2566).

### Animals

The study was conducted at a dog shelter in Mae Taeng District, Chiang Mai Province, Thailand, which ranked highest among eight shelters in a previous welfare study (Shelter A) [32]. The shelter housed 511 healthy dogs and 45 that were disabled due to spinal fractures and dislocations across 110 pens, with an average pen area of 101.9 m² (range: 35–400 m²). The average number of dogs per pen was five (range, 1–18), and the average living space per dog was 20.3 m² (range, 5.83 to 50 m²). Only dogs that had been at the shelter for more than 1 year were eligible for sampling (n = 399 healthy; 43 disabled). For the healthy group, 60 dogs were randomly selected for sample collection, whereas all qualified disabled dogs were sampled.

Disabled dogs were group-housed in a total of eight pens; five pens accommodated five dogs each, and three pens housed six dogs each. All of the dogs were otherwise in good general health, with no signs of systemic disease or illness beyond the physical impairments. None were receiving painkillers or anti-inflammatory medications at the time of sampling. However, five disabled dogs presented with superficial pressure sores (bed sores), which were managed with regular wound cleaning, bandaging, and topical care, but no systemic (oral or injectable) medications. Vitamin B-complex supplements were provided every other day to support neurological health.

The shelter conducted routine pen cleaning twice daily, in the morning and evening (0800 and 1700 hours), and provided food to the dogs at those times. The shelter had an on-site animal hospital, staffed by eight veterinarians and twelve veterinary assistants who were responsible for routine health checks and medical treatment. Additionally, the shelter allowed volunteers and visitors to interact with the dogs through activities such as walking or playtime in designated areas. Enrichment was provided, including toys placed in the pens and access to a swimming pool for dogs to play and relax [32]. Mobility support for disabled dogs was provided through custom wheelchairs to assist ambulation outside their enclosures, ensuring that all dogs were able to go outside and participate in enrichment activities comparable to those of healthy dogs.

### Sample collection

Fecal samples for fGCM determination were collected over a 1-month period in May 2023. Samples were collected between 0600 and 0800 hours using a randomized group-based sampling approach, in which freshly deposited feces were collected at random from the ground within each housing area, without linking samples to individual dogs. Each sample was immediately placed into a sterile plastic zip-lock bag and transferred to a −20°C freezer within 1 hour of collection. For microbiome analysis, 20 samples per group were selected randomly over the 1-month period.

Approximately 5 ml of blood also was collected from either the cephalic (forelimb) or saphenous (hindlimb) vein between 1300 and 1530 hours. Sampling was random and not linked to fecal samples for the 60 healthy dogs. Samples were centrifuged at 1,500 × g for 10 minutes to separate serum, which was stored at −20°C. Dogs were not fasted before blood collection, which occurred 5–6 hours after the first feeding.

### Fecal extraction and fGCM analysis

All chemicals were obtained from Sigma Chemical Company (St. Louis, MO, USA), unless otherwise stated. The fecal hormone extraction method was adapted from Brown et al. [33]. Wet fecal samples were dried in a conventional oven at 60°C for approximately 24–48 hours, then stored at −20°C until hormone extraction. Prior to extraction, dried fecal samples were thawed at room temperature (RT), thoroughly homogenized, and 0.2 g (±0.01 g) of powdered feces was transferred into a glass tube containing 90% ethanol (v/v) in distilled water. Each sample was extracted twice by boiling in a 96°C water bath for 20 minutes, with 100% ethanol added as necessary to prevent drying. After extraction, samples were centrifuged at 1,200 × g for 20 minutes. The resulting supernatants were pooled and evaporated to dryness under a stream of air in a 50°C water bath. Dried extracts were reconstituted in 3 mL of ethanol by vortexing for 1 minute, then re-dried and re-dissolved in methanol with vortexing prior to analysis. Final extracts were stored at −20°C until fGCM analysis.

The fGCM concentrations were determined using a double-antibody enzyme immunoassay (EIA) with a polyclonal rabbit anti-corticosterone antibody (CJM006, Coralie Munro, UC Davis, CA, USA). A 96-well microtiter plate was coated with 150 μL of anti-rabbit IgG (0.01 mg/mL) per well and incubated at RT for 15–24 hours. After incubation, wells were emptied and blotted dry, then blocked with 250 μL of blocking solution and incubated again at RT for 15–24 hours. Plates were subsequently dried at RT (Sanpla Dry Keeper, Sanplatec Corp., Auto A-3, Japan) with loose desiccant until humidity dropped below 20%, then sealed in foil bags with a 1 g desiccant packet and stored at 4°C until use. For the assay, 50 μL of sample extracts and corticosterone standards were added in duplicate to each well, followed by 25 μL of horseradish peroxidase (HRP)-conjugated corticosterone (diluted 1:30,000) and 25 μL of anti-corticosterone antibody (diluted 1:100,000). Plates were incubated at RT for 2 hours, then washed five times with wash buffer. Subsequently, 100 μL of tetramethylbenzidine (TMB) substrate solution was added to each well and incubated at RT for 15–20 minutes. The enzyme reaction was stopped by adding 2 M sulfuric acid (H₂SO₄), and absorbance was measured at 450 nm using a microplate reader (TECAN, Männedorf, Switzerland). The assay sensitivity, based on 90% binding, was 0.14 ng/mL. All samples were analyzed in duplicate. The intra-assay and inter-assay coefficients of variation (CVs) were <10% and 9.4%, respectively.

### Metabolic parameters analysis

Serum MDA concentrations were quantified by a thiobarbituric acid reacting substances (TBARS) assay described by Satitmanwiwat et al. [34]. Briefly, 50 µL of serum and standard were mixed with 750 µL of 0.44 M phosphoric acid, 250 µL of 42 mM thiobarbituric acid (TBA) and 450 µL of distilled water. The mixtures were boiled for 15 min, cooled on ice for 5 min, and centrifuged at 1,500 x g for 5 min. The supernatant was collected and measured an absorbance at 532 nm using UV-VIS spectrophotometer (Shimadzu, Japan). MDA concentrations in samples was calculated from standard curve of MDA equivalents generated by the acid-catalyzed hydrolysis of 1,1,3,3-tetramethoxypropane (TMP) (5–80 µM).

Serum insulin concentrations were measured using the Mercodia Bovine Insulin ELISA kit (Mercodia AB, Uppsala, Sweden), following the manufacturer’s instructions. All reagents and samples were brought to RT prior to use. Enzyme conjugate (1X solution) and wash buffer (1X solution) were prepared by dilution as instructed. Calibrators, controls, and samples (25 µL each) were pipetted in duplicate into microplate wells, followed by 100 µL of enzyme conjugate solution. The plate was incubated on a shaker at 700–900 rpm for 2 hours at room temperature. Wells were then washed six times with 350 µL of wash buffer. Subsequently, 200 µL of TMB substrate was added to each well, and the plate was incubated at room temperature for 15 minutes. The reaction was stopped by adding 50 µL of stop solution (0.5 M H₂SO₄), and the plate was gently shaken for 5 seconds. Optical density was measured at 450 nm using a microplate reader (TECAN, Männedorf, Switzerland).

Serum fructosamine was measured by a colorimetric method using nitroblue tetrazolium in a Biosystems BA400 clinical chemistry analyzer (Biosystems S.A., Barcelona, Spain). Plasma glucose was measured by a glucose oxidase-peroxidase (GOD-POD) method using a Biosystems BA400 clinical chemistry analyzer with quinoneimine measured at 510 nm. Serum lipids were quantified using an Automated Clinical Chemistry Analyzer (Biosystems BA400). TC was measured by a cholesterol oxidase-peroxidase (CHOD-PAP) method. Triglycerides were measured by a colorimetric enzymatic test using glycerol-3-phosphate oxidase-peroxidase (GPO-POD) method, LDL and HDL were measured using homogeneous assay methods.

### DNA extraction and 16S rRNA gene sequencing

Fecal DNA was extracted using the ZymoBIOMICS™ DNA Miniprep Kit (Zymo Research Corporation, CA, USA) following the manufacturer’s protocol. Briefly, 200 mg of feces was added to a ZR BashingBead™ Lysis Tube with 750 μL of lysis solution, then homogenized using a bead beater. Lysates were centrifuged at ≥10,000 × g for 1 minute, and up to 400 μL of supernatant was transferred to a Zymo-Spin™ III-F filter, followed by centrifugation at 8,000 × g for 1 minute. The filtrate was mixed with 1,200 μL of DNA binding buffer, and 800 μL of this mixture was applied twice to a Zymo-Spin™ IICR column, each followed by centrifugation at 10,000 × g for 1 minute. The column was washed sequentially with 400 μL and 700 μL of DNA wash buffer 1 and 2, respectively, then with a final 200 μL wash of DNA wash buffer 2. DNA was eluted by adding 80 μL of DNase/RNase-Free water to the column, incubated for 1 minute, and centrifuged at 10,000 × g. To remove inhibitors, 600 μL of HRC prep solution was passed through a Zymo-Spin™ III-HRC filter, and the eluted DNA was further filtered through this column by centrifugation at 16,000 × g for 3 minutes. The purified DNA was then ready for downstream 16S rRNA gene sequencing. The quality of DNA samples was evaluated using 2% agarose gel electrophoresis. The V4 region of the 16S rRNA gene was amplified using 515F/806R 16S rRNA primers (Novogene Co., Ltd., Beijing, China). The libraries were sequenced on an Illumina paired-end platform (Illumina, San Diego, CA, USA) to generate 300 bp paired-end raw reads, according to the manufacturer’s instructions. All PCR amplification and sequencing steps were carried out at Novogene Co., Ltd. (Beijing, China.) using an Illumina NovaSeq 6000 platform. Sequence reads were analyzed using the Quantitative Insights Into Microbial Ecology 2 (QIIME2) pipeline, specifically version 2022.8. The paired-end sequences were de-noised and merged using the DADA2 plugin within QIIME2 [35,36], which includes quality filtering and trimming of low-quality reads, denoising, merging of paired-end reads, and removal of chimeric sequences, resulting in high-resolution amplicon sequence variants (ASVs). Taxonomic assignment of the 16S rRNA sequences was performed using the Silva 138 99% taxonomy classifier [37,38]. Amplicon sequence variants (ASVs) were aligned using the mafft plugin in QIIME2.

### Statistical analysis

Descriptive data for fGCM, MDA, fructosamine, insulin, glucose, triglycerides, cholesterol, LDL, HDL, and relative abundance of gut microbiota are presented as mean ± standard error of the mean (SEM). Statistical analyses were performed using R software [39]. To assess differences in biomarker concentrations between healthy and disabled dogs, the distribution of each variable was first evaluated for normality using the Shapiro–Wilk test [40], and homogeneity of variances was assessed using Levene’s test. For variables that satisfied both the normality and homogeneity of variance assumptions (MDA, fructosamine, and HDL), group comparisons were performed using the independent samples Student’s *t*-test. For variables that violated at least one of these assumptions (fGCM, insulin, glucose, triglycerides, total cholesterol, and LDL), the non-parametric Wilcoxon rank-sum test was applied. Statistical significance was set at *p* < 0.05. To assess microbial diversity, alpha diversity indices, including Observed features, Shannon entropy, and Pielou evenness, were calculated. Statistical comparisons of alpha diversity between groups were performed using the Mann-Whitney test. The relative abundances of microbial taxa at the phylum, family, and genus levels were described using descriptive statistics and presented in percentages, and group comparisons were performed using the Wilcoxon rank-sum test. For beta diversity analysis, the Bray-Curtis, Jaccard, Unweighted UniFrac and Weighted UniFrac distance matrix were used, and the results were visualized through principal coordinate analysis (PCoA) in R software v4.4.1. Permutational Multivariate Analysis of Variance (PERMANOVA) was used to statistically test for significant differences in microbial composition between the two groups.

## 3. Results

### fGCM and metabolic markers

Descriptive data for fGCM, MDA, fructosamine, insulin, glucose, triglycerides, cholesterol, LDL, and HDL are summarized in Table 1. Mean fGCM concentrations did not differ between healthy and disabled dogs. In contrast, disabled dogs exhibited significantly higher MDA and glucose levels and lower triglyceride and HDL concentrations than healthy dogs (*p* < 0.05 for all comparisons). Additionally, no differences were observed for fructosamine (*p* = 0.6437), insulin (*p* = 0.9413), cholesterol (*p* = 0.0644), or LDL (*p* = 0.2661) concentrations.

**Table 1:** Mean ± standard error (SE) and range of physiological and metabolic parameters measured in healthy and disabled dogs.

Parameters	Healthy Dogs (n = 60)	Disabled Dogs (n = 43)	P-value
fGCM (ng/g)	188.70 ± 8.21 (92.57–697.99)	175.77 ± 9.52 (40.33–443.26)	0.2524
MDA (µM)	7.80 ± 0.1 (1.89–15.66)	9.60 ± 0.47 (1.51–16.98)	0.0022
Fructosamine (mM)	1.29 ± 0.02 (0.99–1.68)	1.32 ± 0.02 (0.98–1.79)	0.6437
Insulin (μg/L)	736.68 ± 92.32 (0.00–4255.00)	693.67 ± 59.06 (0.00–2156.00)	0.9413
Glucose (mg/dL)	63.00 ± 2.50 (21.00–135.00)	70.83 ± 2.16 (30.00–116.00)	0.0357
Triglyceride (mg/dL)	95.45 ± 6.33 (11.00–256.00)	65.67 ± 3.34 (23.00–134.00)	0.0002
Cholesterol (mg/dL)	179.53 ± 5.80 (92.00–363.00)	164.52 ± 4.85 (71.00–248.00)	0.0644
LDL (mg/dL)	4.75 ± 0.52 (0.00–23.00)	5.48 ± 0.42 (0.00–14.00)	0.2661
HDL (mg/dL)	141.40 ± 3.78 (74.00–198.00)	122.50 ± 3.39 (54.00–178.00)	<0.0001

fGCM = fecal glucocorticoid metabolites; MDA = malondialdehyde; LDL = low-density lipoprotein; HDL = high-density lipoprotein.

### Gut microbial diversity

**Fig 1:**
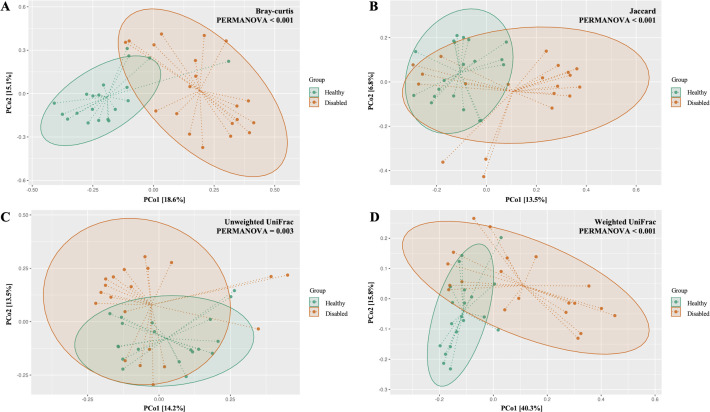
Principal Coordinates Analysis (PCoA) of fecal microbiota beta diversity in healthy and disabled dogs. PCoA plots showing beta diversity of fecal microbiota in healthy (green) and disabled (orange) dogs based on four distance metrics: (A) Bray-Curtis, (B) Jaccard, (C) Unweighted UniFrac, and (D) Weighted UniFrac. Each point represents a fecal sample; ellipses indicate 95% confidence intervals. Distinct clustering patterns reflect compositional differences. PERMANOVA revealed significant differences across all metrics.

No significant differences were observed between healthy and disabled dogs in alpha diversity of gut microbiota assessed using Observed features, Shannon entropy, and Pielou evenness. By contrast, there were differences in beta diversity of gut microbial community composition, as assessed by Bray-Curtis, Jaccard, Unweighted UniFrac, and Weighted UniFrac distance metrics (PERMANOVA: Bray–Curtis R² = 0.12, p < 0.001; Jaccard R² = 0.07, p < 0.001; Unweighted UniFrac R² = 0.06, p = 0.003; Weighted UniFrac R² = 0.18, p < 0.001). PERMDISP indicated no significant differences in within-group dispersion for Bray–Curtis, Jaccard, or Unweighted UniFrac (p = 0.754, 0.884, and 0.781, respectively), whereas dispersion differed significantly between groups for Weighted UniFrac (p = 0.016) (Fig 1).

**Fig 2:**
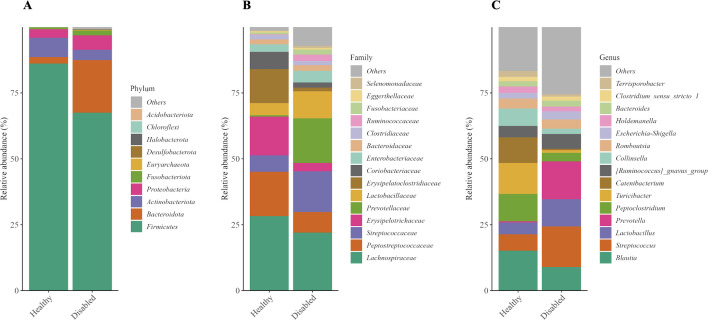
Taxonomic composition of fecal microbiota in healthy and disabled dogs. (A) phylum, (B) family, and (C) genus levels based on 16S rRNA gene sequencing. Bars represent the average relative abundance for each group. At the phylum level, both groups were dominated by Firmicutes and Bacteroidetes. At the family and genus levels, healthy dogs showed greater abundance of Lachnospiraceae, Peptostreptococcaceae, Erysipelotrichaceae, *Blautia*, and *Peptoclostridium*, while disabled dogs exhibited higher levels of Prevotellaceae, Streptococcaceae, *Prevotella*, *Lactobacillus*, and *Streptococcus.*

Multiple bacterial phyla and genera were initially detected across all amplicon sequence variants (ASVs) in the fecal microbiota. However, only five major phyla: Firmicutes, Bacteroidetes, Actinobacteria, Proteobacteria, and Fusobacteria accounted for the vast majority of the total microbial community and were therefore used for downstream ecological interpretation. Compositional analysis revealed that gut microbial communities in both healthy and disabled dogs were predominantly composed of these five phyla, although relative abundances differed between groups. P-values for relative abundance comparisons were not adjusted for multiple testing and are therefore considered exploratory. At the phylum level, Firmicutes dominated in both groups; however, relative abundance was markedly higher in healthy compared to disabled dogs. In contrast, Bacteroidetes was substantially more abundant in disabled dogs. Additionally, healthy dogs exhibited a higher relative abundance of Actinobacteria, whereas Proteobacteria was more prevalent in disabled dogs, suggesting potential alterations in gut microbial composition associated with health status. Furthermore, Fusobacteria was also more abundant in disabled dogs compared to healthy dogs (Table 2, Fig 2).

**Table 2:** Relative abundance (%) of the five most common bacterial taxa at the phylum, family, and genus levels in healthy and disabled dogs.

Taxa	Healthy Dogs (n = 20)	Disable Dogs (n = 20)	P*-*value
**Phyla**			
Firmicutes	86.15 ± 2.78 (55.55–99.48)	67.53 ± 4.84 (28.54–99.96)	0.0023
Bacteroidetes	2.53 ± 0.86 (0.00–14.76)	19.94 ± 4.60 (0.00–66.80)	0.0179
Actinobacteria	7.27 ± 1.41 (0.13–25.00)	3.99 ± 1.05 (0.00–19.91)	0.0524
Proteobacteria	3.26 ± 2.08 (0.01–35.43)	5.38 ± 1.67 (0.02–30.09)	0.0263
Fusobacteria	0.73 ± 0.38 (0.00–6.76)	1.87 ± 0.68 (0.00–13.27)	0.1292
Other	0.05 ± 0.02 (0.00–0.45)	1.34 ± 0.97 (0.00–17.99)	0.1836
**Family**			
Lachnospiraceae	28.33 ± 4.28 (0.56–64.59)	22.05 ± 5.09 (0.15–83.79)	0.2012
Peptostreptococcaceae	16.73 ± 3.27 (2.83–65.76)	7.82 ± 2.21 (0.12–39.34)	0.0022
Streptococcaceae	6.24 ± 2.80 (0.05–42.14)	15.47 ± 4.07 (0.02–65.32)	0.0223
Erysipelotrichaceae	14.69 ± 5.61 (0.01–88.29)	3.09–0.78 (0.00–12.35)	0.2211
Prevotellaceae	0.50 ± 0.33 (0.00–6.71)	16.96 ± 4.35 (0.00–63.79)	0.0026
Other	33.49 ± 4.23 (1.59–82.32)	34.58 ± 4.86 (0.15–80.77)	0.2465
**Genera**			
*Blautia*	15.06 ± 2.71 (0.10–35.99)	8.97 ± 2.36 (0.09–42.99)	0.0859
*Streptococcus*	6.23 ± 2.80 (0.05–42.13)	15.40 ± 4.05 (0.02–65.10)	0.0223
*Lactobacillus*	4.55 ± 2.22 (0.00–41.06)	10.23 ± 3.81 (0.01–76.15)	0.0583
*Prevotella*	0.34 ± 0.29 (0.00–5.81)	14.39 ± 4.01 (0.00–61.55)	0.0015
*Peptoclostridium*	10.47 ± 1.81 (0.43–27.58)	3.32 ± 0.84 (0.01–11.96)	0.0014
Other	63.34 ± 4.72 (28.35–96.62)	47.67 ± 4.94 (7.81–96.24)	0.0664

**Fig 3:**
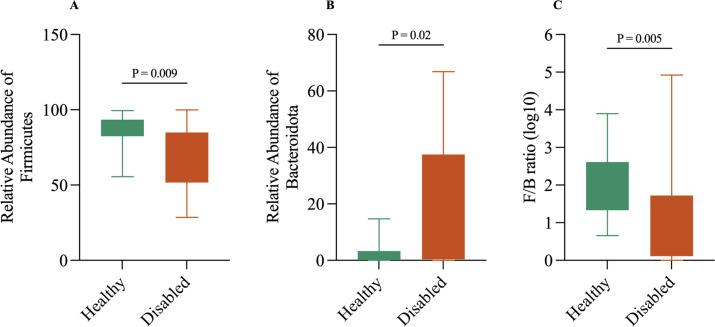
Relative abundance of major bacterial phyla and Firmicutes-to-Bacteroidetes (F/B) ratio in healthy and disabled dogs. Box-and-whisker plots show the distribution of (A) Firmicutes, (B) Bacteroidetes, and (C) the log₁₀-transformed Firmicutes-to-Bacteroidetes (F/B) ratio. Boxes represent the interquartile range (IQR), and whiskers denote the minimum and maximum values. Firmicutes abundance was significantly lower in disabled dogs (*p* = 0.009), whereas Bacteroidetes abundance was significantly higher (*p* = 0.020). The F/B ratio was also significantly reduced in disabled dogs (*p* = 0.005). Statistical comparisons between groups were performed using the Mann–Whitney U test.

In the present study, healthy dogs exhibited a markedly higher Firmicutes-to-Bacteroidetes (F/B) ratio compared to disabled dogs, indicating a pronounced difference in microbial composition between the groups (Table 3). This pattern was statistically significant when log10-transformed F/B ratios were compared (*p* = 0.005) (Fig 3). The underlying relative abundance data revealed that Firmicutes were more prevalent in healthy dogs than in disabled dogs, whereas Bacteroidetes were more abundant in disabled dogs compared to healthy counterparts.

**Table 3:** Relative abundance of Firmicutes and Bacteroidetes phyla and the Firmicutes-to-Bacteroidetes (F/B) ratio in healthy and disabled dogs.

Group	Firmicutes (%)	Bacteroidetes (%)	F/B ratio
Healthy dogs (n = 20)	86.15^b^	2.53^a^	34.05^b^
Disabled dogs (n = 20)	67.53^a^	19.98^b^	3.38^a^

At the family level, Lachnospiraceae, Peptostreptococcaceae, and Erysipelotrichaceae were the predominant taxa in healthy dogs, whereas Lachnospiraceae, Streptococcaceae, and Prevotellaceae were the dominant families identified in disabled dogs. Notably, the relative abundances of Peptostreptococcaceae and Erysipelotrichaceae were markedly reduced in disabled dogs compared to healthy dogs. In contrast, Prevotellaceae were more prevalent in disabled dogs than in healthy counterparts. At the genus level, *Blautia* was the most prevalent in healthy dogs but was reduced in disabled dogs. Conversely, *Streptococcus* and *Lactobacillus* were more abundant in disabled than in healthy dogs. In addition, *Prevotella*, a genus typically associated with carbohydrate metabolism, was markedly increased in disabled compared to healthy dogs. In contrast, *Peptoclostridium* was more abundant in healthy than in disabled dogs. These compositional shifts suggest notable differences in gut microbial community structure between the two groups (Table 2, Fig 2).

## 4. Discussion

This study investigated physiological stress, metabolic parameters, and gut microbiota composition in healthy and disabled shelter dogs housed under standardized conditions and management at a large dog shelter in Thailand. The results revealed no significant differences in fGCM concentrations between the two groups, while disabled dogs exhibited metabolic and microbial imbalances possibly linked to oxidative stress and dysbiosis, and might reflect heightened physiological demands of chronic disability. The shelter in this study participated in a companion study, and among the eight shelters evaluated, was ranked highest in welfare indicators [32] and also had dogs with the lowest fGCM concentrations (unpublished). It also was the only shelter in the area that specialized in caring for disabled dogs.

In other shelter environments, elevated fGCM concentrations have been reported in dogs experiencing poor housing conditions, limited enrichment, or social isolation, and correlated with behavioral signs of distress such as excessive barking, stereotypies, and reduced social behaviors [5,41,42]. By contrast, environmental enrichment, high-quality care, and social support within shelter environments can effectively reduce stress in dogs as evidenced by both behavioral improvements and reductions in fGCM concentrations [5,43,44]. The absence of higher fGCM in disabled dogs compared to healthy controls in our study is noteworthy and suggests that the quality of care, environmental enrichment, and social support provided may be sufficient to buffer the physiological impacts of physical impairments.

While disabled dogs experience physiological limitations associated with reduced mobility and chronic impairment [45], our findings suggest that structured management protocols and environmental predictability, such as consistent feeding schedules, routine cleaning, stable enclosure layouts, and regular human–animal interactions, can play a role in supporting physiological welfare under long-term shelter conditions. These factors may help buffer stress responses in disabled dogs despite their reduced capacity to regulate stress through physical activity. Previous studies in dogs have demonstrated that regular routines, positive human interaction, and environmental complexity can lower fGCM concentrations, even in confined settings [46,47]. Cross-species evidence supports this pattern: for example, captive leopard cats (*Felis bengalensis*) housed near large predators, such as lions and tigers, showed chronically elevated cortisol levels; however, stress was alleviated through environmental enrichment, including the provision of hiding places and vegetation [48]. Likewise, physically impaired primates housed in enriched environments did not exhibit sustained increases in adrenal activity [49].

It is important to recognize that fGCM measures can reflect cumulative stress over time and may not capture acute or transient stressors, such as sudden environmental changes, unfamiliar human interactions, or brief social conflicts. While our study design accounted for long-term housing and standardized care, subtle episodic stressors may not be detected solely through fGCM measurements. Furthermore, individual variability in HPA responsiveness, metabolic clearance rates, or gut transit time may influence fecal hormone concentrations and could mask small group-level effects [50,51]. Nonetheless, our findings provide evidence that when shelters implement comprehensive welfare practices, including veterinary care, socialization opportunities, and environmental enrichment, dogs with physical disabilities can achieve comparable stress hormone profiles to their healthy peers. These outcomes reinforce the importance of management quality in determining welfare trajectories in shelter populations, and they emphasize that physical disability alone should not be presumed to equate to poor welfare.

Although no significant differences in fGCM concentrations were observed between healthy and disabled dogs, several metabolic parameters differed significantly, providing additional insight into the physiological status of that group. Specifically, disabled dogs exhibited higher concentrations of MDA and glucose, while levels of triglycerides and HDL were lower compared to healthy counterparts. These biomarkers are closely linked to metabolic function, oxidative stress, and lipid homeostasis, and together may reflect subtle yet important differences in health and welfare between the two groups.

MDA is a byproduct of lipid peroxidation and is widely used as a marker of oxidative stress [52]. Elevated MDA concentrations in disabled dogs suggest increased oxidative damage, which could stem from chronic inflammation, impaired mitochondrial function, or reduced physical activity, all of which were commonly associated with mobility impairment [53]. Similar trends have been reported in other species with compromised physical conditions. For example, studies in sick elephants have shown that MDA concentrations were approximately 30% higher than those in healthy individuals [54]. In dogs, prolonged confinement was also associated with oxidative stress, likely due to increased lipid peroxidation and alterations in antioxidant enzyme activities [55]. Although short-term, that study highlights how stressors involving confinement and restricted movement can elevate oxidative stress markers, emphasizing the need for mobility-supportive environments in shelters.

The observation of higher serum glucose in disabled dogs may indicate alterations in energy metabolism or reduced insulin sensitivity. Although insulin and fructosamine concentrations did not differ significantly between groups, elevated glucose levels may reflect metabolic imbalance. Chronic stress and impaired locomotion, as observed in physically disabled dogs, disrupt glucose homeostasis by stimulating hepatic gluconeogenesis and reducing glucose uptake in peripheral tissues [56]. In companion animals, particularly dogs, such changes may predispose individuals to metabolic disorders over time. It is noteworthy that despite similar fGCM concentrations, these metabolic shifts may represent more subtle or localized physiological responses to stress or reduced activity.

The lower concentrations of triglycerides and HDL observed in disabled dogs may indicate alterations in lipid metabolism associated with restricted mobility. In healthy animals, adequate physical activity and a balanced diet support normal lipid synthesis and turnover [57]. Reduced lipid concentrations in disabled dogs could therefore be partly attributed to decreased physical activity due to their condition. Previous studies in companion dogs showed that lipid metabolism was influenced by factors such as age and body weight, with lower triglyceride concentrations often associated with reduced activity levels [58]. Moreover, research on dogs with inflammatory diseases demonstrated that metabolic disruptions and illness can lead to declines in cholesterol and HDL [59], further supporting the link between reduced physical function and altered lipid profiles in disabled dogs. Notably, HDL, often termed “protective” cholesterol, plays a key role in anti-inflammatory processes and vascular health [60]. A reduction in HDL may therefore reflect not only impaired lipid metabolism but also increased vulnerability to chronic inflammation or cardiovascular risk, even in the absence of clinical symptoms [61]. Similar patterns have been reported in elderly or immobile animals, where reduced HDL and cholesterol concentrations were associated with systemic aging and physiological stress [62], Additionally, dogs with inflammatory protein-losing enteropathy, a chronic intestinal condition, exhibited significantly lower HDL and higher C-reactive protein concentrations compared to healthy controls, suggesting that reduced HDL is linked to systemic inflammation [59]. These findings suggest that the metabolic alterations observed in disabled shelter dogs may mirror those observed in other contexts of physical limitation or chronic stress.

Microbiome analyses revealed substantial differences in gut microbial composition between healthy and disabled shelter dogs, providing critical insights into the potential link between host physical status and intestinal health. Although alpha diversity metrics did not differ significantly between groups, indicating similar species richness and evenness within individuals, this does not necessarily reflect functional similarity or equivalence in health status. Indeed, previous studies in dogs and other mammals have shown that alpha diversity alone is not a reliable predictor of gut health, as individuals with normal microbial richness may still exhibit dysbiosis if the community composition is significantly altered [17,63,64]. In contrast, beta diversity analyses, which evaluate inter-individual differences in community structure [65], revealed clear and statistically significant separation between healthy and disabled dogs across all metrics. This suggests that although the microbial richness was conserved, the overall microbial community structure and phylogenetic composition were significantly reshaped in the disabled group, potentially reflecting physiological or immunological adaptations associated with chronic physical impairment [66,67].

One of the most striking findings was the significantly reduced Firmicutes-to-Bacteroidetes (F/B) ratio in disabled dogs. This ratio is frequently cited as a marker of intestinal health and metabolic state [68]. In dogs, a higher F/B ratio is often associated with stable gut homeostasis, whereas a reduced ratio has been linked to dysbiosis, inflammation, and altered metabolic processing [69]. Our findings align with patterns observed in dogs experiencing stress, obesity, or gastrointestinal disorders, in which a decrease in Firmicutes and a corresponding increase in Bacteroidetes have been indicative of compromised mucosal integrity or shifts in dietary fermentation pathways [69]. While direct evidence in dogs is scarce, rodent and human studies consistently showed that conditions of injury, immobility, or chronic stress lead to reduced F/B ratios, lower overall short-chain fatty acid production (especially butyrate), and heightened inflammatory markers, hallmarks of dysbiosis and impaired gut metabolic function [70–72]. The elevated abundance of Proteobacteria and Fusobacteria in disabled dogs further underscores the possibility of a pro-inflammatory or stress-affected microbial milieu, as these phyla are often enriched under inflammatory conditions or during microbial instability [73,74].

At the family and genus levels, taxonomic shifts further indicated alterations in gut microbial ecology among dogs with disabilities. Beneficial commensal taxa such as Peptostreptococcaceae and Erysipelotrichaceae, which is typically associated with butyrate production, mucosal protection, and immune tolerance [75,76], were markedly lower in the disabled group. Butyrate-producing bacteria such as *Peptoclostridium,* play essential roles in maintaining gut epithelial integrity and regulating T-cell-mediated inflammation [77]. Their depletion has been associated with conditions including irritable bowel syndrome, metabolic syndrome, and chronic stress [78,79]. In contrast, disabled dogs showed elevated concentrations of Prevotellaceae, Lactobacillaceae, and Streptococcaceae. Although *Prevotella* is generally associated with carbohydrate fermentation and mucosal immune stimulation in healthy gut environments [80], its marked overrepresentation in the disabled group observed in our study may reflect an unfavorable shift in microbial balance. Previous research has linked high *Prevotella* abundance to increased mucin degradation and elevated intestinal epithelial permeability factors, which may influence gut barrier function under certain conditions [81]. Furthermore, while *Streptococcus* is a typical component of the gut microbiota, it can behave opportunistically in some inflammatory or immunocompromised contexts [82]. The increased abundance of this genus observed in disabled dogs may therefore reflect altered mucosal conditions or immune–microbial interactions, rather than indicating pathology. Interestingly, increased *Lactobacillus*, often considered beneficial [83], may also represent a compensatory microbial response or shifts in lactic acid fermentation, as reported in rodent models [84,85]. Taken together, these microbial patterns suggest that, even in the absence of overt clinical symptoms, disabled dogs may harbor a gut microbiota that is functionally distinct, although the physiological implications of these compositional differences remain to be fully elucidated. Comparative studies in other species reinforce these interpretations. In primates and rodents, physical impairment or chronic illness has been linked to lower microbial diversity and shifts toward pro-inflammatory microbial taxa. For instance, studies in mice with induced hindlimb unloading have demonstrated gut dysbiosis characterized by an increase in Bacteroidetes and a decrease in Firmicutes [86], findings similar to ours. Likewise, sedentary humans often exhibit reduced butyrate‐producing taxa and lower microbial stability compared to more active individuals [87], and companion dogs with chronic mobility‐limiting conditions demonstrated decreased key butyrate producers and greater community variability [88].

This study has limitations, most notably the small sample sizes for microbiota analyses that may limit the generalizability of microbial findings. The cross-sectional design also restricts causal inference. In addition, behavioral assessments and immunological markers, such as secretory immunoglobulin A or stress leukogram patterns, were not included [89–92], yet could provide complementary welfare insights. Future studies should adopt a longitudinal design and include additional measures of body condition, muscle mass, inflammatory cytokines, short-chain fatty acid profiles, and metagenomics, as well as behavioral responses, to better understand the long-term welfare implications of physical disability in confined environments.

## 5. Conclusions

The findings of this study indicate that disabled dogs housed in a well-managed shelter environment did not exhibit elevated fGCM concentrations but showed subtle physiological alterations reflected in metabolic and gut microbial parameters. These results suggest that stress hormone measures alone may not fully capture welfare status in dogs with physical impairments, and that a multidimensional assessment incorporating metabolic and microbial markers may provide a more sensitive evaluation of health and welfare in special-needs populations.

While alpha diversity did not differ between groups, differences in beta diversity and overall taxonomic composition suggest variation in gut microbiota structure between healthy and disabled dogs; however, these findings should be interpreted cautiously as exploratory. Overall, our results support the capacity of high-standard shelters to maintain general welfare while highlighting the importance of tailored physiological monitoring for disabled dogs.

## Supporting information

S1 Table Sample metadata and NCBI Sequence Read Archive (SRA) accession numbers for all fecal samples included in this study. (DOCX)
