# Supplementary material for: Comparative analysis of stress markers, metabolic health, and gut microbiota in healthy and disabled dogs in long-term shelters in Thailand
Source: PLoS One. 2026 Mar 13;21(3):e0344383. doi: 10.1371/journal.pone.0344383 (PMC12987422; doi:10.1371/journal.pone.0344383)
Supplement: S1 Table — (DOCX) [file pone.0344383.s001.docx]

The raw sequencing data generated from this study is available under BioProject ID: PRJNA1287447 and PRJNA1427372 at GenBank (Supplement table S1). (<https://www.ncbi.nlm.nih.gov/bioproject/PRJNA1427372> and <https://www.ncbi.nlm.nih.gov/bioproject/PRJNA1287447>)

**Supplement table S1**. Sample metadata and SRA accessions used in this study

| Sanple ID | Group | BioProject | BioSample | SRA Accession |
| --- | --- | --- | --- | --- |
| EH21 | Healthy | PRJNA1287447 | SAMN49820330 | SRR34394528 |
| EH32 | Healthy | PRJNA1287447 | SAMN49820331 | SRR34394527 |
| EH38 | Healthy | PRJNA1287447 | SAMN49820332 | SRR34394516 |
| EH49 | Healthy | PRJNA1287447 | SAMN49820333 | SRR34394505 |
| EH57 | Healthy | PRJNA1287447 | SAMN49820334 | SRR34394494 |
| EH93 | Healthy | PRJNA1287447 | SAMN49820335 | SRR34394483 |
| EH101 | Healthy | PRJNA1287447 | SAMN49820336 | SRR34394472 |
| EH129 | Healthy | PRJNA1287447 | SAMN49820337 | SRR34394471 |
| EH145 | Healthy | PRJNA1287447 | SAMN49820338 | SRR34394470 |
| EH180 | Healthy | PRJNA1287447 | SAMN49820339 | SRR34394469 |
| EH182 | Healthy | PRJNA1287447 | SAMN49820340 | SRR34394526 |
| EH198 | Healthy | PRJNA1287447 | SAMN49820341 | SRR34394525 |
| EH209 | Healthy | PRJNA1287447 | SAMN49820342 | SRR34394524 |
| EH211 | Healthy | PRJNA1287447 | SAMN49820343 | SRR34394523 |
| EH261 | Healthy | PRJNA1287447 | SAMN49820344 | SRR34394522 |
| EH277 | Healthy | PRJNA1287447 | SAMN49820345 | SRR34394521 |
| EH278 | Healthy | PRJNA1287447 | SAMN49820346 | SRR34394520 |
| EH281 | Healthy | PRJNA1287447 | SAMN49820347 | SRR34394519 |
| EH282 | Healthy | PRJNA1287447 | SAMN49820348 | SRR34394518 |
| EH294 | Healthy | PRJNA1287447 | SAMN49820349 | SRR34394517 |
| ED02 | Disabled | PRJNA1427372 | SAMN55915729 | SRR37338170 |
| ED04 | Disabled | PRJNA1427372 | SAMN55915730 | SRR37338169 |
| ED08 | Disabled | PRJNA1427372 | SAMN55915731 | SRR37338158 |
| ED14 | Disabled | PRJNA1427372 | SAMN55915732 | SRR37338157 |
| ED15 | Disabled | PRJNA1427372 | SAMN55915733 | SRR37338156 |
| ED18 | Disabled | PRJNA1427372 | SAMN55915734 | SRR37338155 |
| ED19 | Disabled | PRJNA1427372 | SAMN55915735 | SRR37338154 |
| ED21 | Disabled | PRJNA1427372 | SAMN55915736 | SRR37338153 |
| ED32 | Disabled | PRJNA1427372 | SAMN55915737 | SRR37338152 |
| ED33 | Disabled | PRJNA1427372 | SAMN55915738 | SRR37338151 |
| ED41 | Disabled | PRJNA1427372 | SAMN55915739 | SRR37338168 |
| ED45 | Disabled | PRJNA1427372 | SAMN55915740 | SRR37338167 |
| ED48 | Disabled | PRJNA1427372 | SAMN55915741 | SRR37338166 |
| ED52 | Disabled | PRJNA1427372 | SAMN55915742 | SRR37338165 |
| ED57 | Disabled | PRJNA1427372 | SAMN55915743 | SRR37338164 |
| ED58 | Disabled | PRJNA1427372 | SAMN55915744 | SRR37338163 |
| ED59 | Disabled | PRJNA1427372 | SAMN55915745 | SRR37338162 |
| ED60 | Disabled | PRJNA1427372 | SAMN55915746 | SRR37338161 |
| ED62 | Disabled | PRJNA1427372 | SAMN55915747 | SRR37338160 |
| ED64 | Disabled | PRJNA1427372 | SAMN55915748 | SRR37338159 |
